# Limited accessibility to HIV services for persons with disabilities living with HIV in Ghana, Uganda and Zambia

**DOI:** 10.7448/IAS.19.5.20829

**Published:** 2016-07-20

**Authors:** Waimar Tun, Jerry Okal, Katie Schenk, Selina Esantsi, Felix Mutale, Rita Kusi Kyeremaa, Edson Ngirabakunzi, Hilary Asiah, Charlotte McClain-Nhlapo, Grimond Moono

**Affiliations:** 1 HIV and AIDS Program, Population Council, Washington, DC, USA; 2 HIV and AIDS Program, Population Council, Nairobi, Kenya; 3 Department of Global and Community Health, George Mason University, Fairfax, VA, USA; 4 Reproductive Health Program, Population Council, Accra, Ghana; 5 Development Consultant, Formerly with Zambia Agency for Persons with Disabilities, Lusaka, Zambia; 6 Ghana Federation of Disability Organisations, Accra, Ghana; 7 National Union of Disabled Persons of Uganda, Kampala, Uganda; 8 The World Bank, Washington, DC, USA; 9 HIV and AIDS Program, Population Council, Lusaka, Zambia

**Keywords:** disability, persons with disabilities, PLHIV, HIV positive, accessibility

## Abstract

**Introduction:**

Knowledge about experiences in accessing HIV services among persons with disabilities who are living with HIV in sub-Saharan Africa is limited. Although HIV transmission among persons with disabilities in Africa is increasingly acknowledged, there is a need to bring to life the experiences and voices from persons with disabilities living with HIV to raise awareness of programme implementers and policy makers about their barriers in accessing HIV services. This paper explores how the barriers faced by persons with disabilities living with HIV impede their ability to access HIV-related services and manage their disease.

**Methods:**

We conducted focus group discussions with 76 persons (41 females; 35 males) with physical, visual and/or hearing impairments who were living with HIV in Ghana, Uganda and Zambia (2012–2013). We explored challenges and facilitators at different levels (individual, psychosocial and structural) of access to HIV services. Transcripts were analyzed using a framework analysis approach.

**Results:**

Persons with disabilities living with HIV encountered a wide variety of challenges in accessing HIV services. Delays in testing for HIV were common, with most waiting until they were sick to be tested. Reasons for delayed testing included challenges in getting to the health facilities, lack of information about HIV and testing, and HIV- and disability-related stigma. Barriers to HIV-related services, including care and treatment, at health facilities included lack of disability-friendly educational materials and sign interpreters, stigmatizing treatment by providers and other patients, lack of skills to provide tailored services to persons with disabilities living with HIV and physically inaccessible infrastructure, all of which make it extremely difficult for persons with disabilities to initiate and adhere to HIV treatment. Accessibility challenges were greater for women than men due to gender-related roles. Challenges were similar across the three countries. Favourable experiences in accessing HIV services were reported in Uganda and Zambia, where disability-tailored services were offered by non-governmental organizations and government facilities (Uganda only).

**Conclusions:**

Persons with disabilities living with HIV encounter many challenges in accessing HIV testing and continued care and treatment services. Changes are needed at every level to ensure accessibility of HIV services for persons with disabilities.

## Introduction

Persons with disabilities represent approximately 15% of the world's population with 80% living in low- and middle-income countries [1]. Further, evidence indicates that persons with disabilities are at the same or elevated risk of HIV because of the many vulnerabilities they face, including poverty, lack of education, lack of sex education, lack of knowledge about HIV and safe sex practices, sexual violence, substance abuse, poor access to health services and stigma and discrimination [2–11]. A systematic review by De Beaudrap *et al*. found that persons with disabilities do not have a lower risk of HIV infection compared with the general population [12].

To address the needs of persons with disabilities living with HIV, HIV services must be inclusive, addressing their specific needs to ensure early diagnosis and timely initiation of HIV treatment, and promote retention and adherence in care and treatment. While research on the challenges encountered by persons with disabilities in accessing health services in sub-Saharan Africa is growing [2,4,9,11,13–18], crucial practical information on their specific challenges and facilitators remains limited, particularly based on data collection directly from persons with disabilities living with HIV [15,18]. Understanding the unique experiences of persons with disabilities living with HIV from their own perspectives and experiences in accessing HIV services will help programmes to address their specific needs. The objective of this paper is to understand how the barriers faced by persons with disabilities living with HIV impede their ability to access HIV-related services and manage their disease.

## Methods

We conducted a three-country study (Ghana, Uganda and Zambia), representing settings of different stages of the HIV epidemic and the degree to which the needs of persons with disabilities are recognized in the National Strategic Plan for HIV and AIDS (Uganda: high; Zambia: moderate; Ghana: low) [19–22]. In order to explore factors affecting access to and use of HIV services, we conducted focus group discussions (FGD) with persons with disabilities living with HIV (2012–2013). Study activities were conducted in the capital city (Accra, Kampala and Lusaka) and one peri-urban or rural site (Amasaman, Jinja and Solwezi) in each country. We established and sought advice from an advisory board in each country, which consisted of leaders from local disabled persons organizations (DPOs) and country AIDS Commissions to provide guidance on study design, implementation and results interpretation. We conducted FGDs to generate richer discussions based on people's potentially differing experiences; feedback from the advisory board indicated it would be acceptable and appropriate.

A total of 17 FGDs were conducted (median: six per group; IQR: 2.5–6). Participants were 18 years or older, HIV positive and had visual, hearing or physical disabilities. Disability and HIV status were self-reported. Participants were recruited as a convenience sample through DPOs and peer referrals. Designated DPO staff recruited candidates in a private and confidential manner providing information about the study including the eligibility criteria. DPO staff instructed interested candidates to attend the FGD at the specified time and place, where the candidates were screened and consented by a study staff. Participants were also asked to invite potentially interested and eligible peers with disabilities to contact the DPO staff. We recruited individuals accessing and those not accessing services at the DPOs. Persons with intellectual or developmental disabilities were not included as it would have required special procedures for appropriate consent, which the ethical review boards were not comfortable with. Participants and their assistants received reimbursement for time and transport. Assistants waited in another room to maintain confidentiality of participants during the FGD. Trained moderators sensitized in working with persons with disabilities conducted the FGD using a semi-structured guide designed to elicit information about barriers and facilitators to access HIV services. Sign interpreters were used for FGDs with hearing-impaired persons. Before each FGD, a researcher (along with a sign interpreter for deaf participants) sought informed consent individually with each potential participant in private and obtained signature or finger/toe print. Interviews were recorded, transcribed and translated.

FGD transcripts were imported into ATLAS.ti v5.2 (ATLAS.ti GmbH, Berlin, Germany). The research team reviewed transcripts and conducted analysis using a framework analysis approach [23–25], which is appropriate for applied research in order to describe and interpret what is happening in a specific setting to provide recommendations as opposed to generating theory to be tested. Codes were developed using key domains outlined *a priori* during research design; during data analysis, three researchers reviewed the transcripts and added codes based on emergent themes. Themes were assessed and compared to determine how often the same concept emerged within and across countries and by disability type. Analysts double coded 30% of the transcripts to ensure quality.

The study was approved by the ethical review boards of the Population Council, University of Zambia, Ghana Health Services, The AIDS Support Organization-Uganda and the Uganda National Council for Science and Technology.

## Results

We recruited a total of 76 persons with disabilities living with HIV (41 females; 35 males). Table 1 shows the characteristics of the FGD participants. All but two participants had their disabilities prior to their HIV diagnosis.

**Table 1:** Characteristics of focus group participants

	Ghana (*N*=14)	Uganda (*N*=28)	Zambia (*N*=34)
Type of impairment			
Hearing	4	1	8
Visual	1	14	9
Physical	9	12	17
Physical and visual	0	1	0
Sex			
Female	10	16	15
Male	4	12	19
Median age, years (IQR)	43 (36, 48)	40 (34, 50)	39 (30, 47)
Education			
<Primary or none	2	6	3
Completed primary	2	3	6
Completed secondary	8	5	24
Completed high school	1	13	1
>High school	1	1	0
Marital status			
Single	7	7	14
Married	3	14	10
Divorced/widowed/separated	4	7	10

IQR: interquartile range.

### Barriers to HIV testing

Most participants indicated that they did not test for HIV until they became sick; hence, late HIV diagnosis was common among this population, regardless of sex, disability type or country. Most participants reported that they were aware of persons with disabilities who delayed HIV testing until they were critically ill.

… if I hadn't gotten sick and been admitted, I wouldn't have been tested. (Female, blind, 39, Ghana)

One of the primary factors impeding access to testing was the lack of information about HIV and HIV testing. In all three countries, the majority of participants reported being disappointed with the limited amount of information in accessible formats (e.g. Braille, large print and sign interpreters) about HIV and the importance of testing. Other major factors impeding access to HIV testing include limited mobility, lack of transportation, social isolation and HIV- and disability-related stigma (discussed in subsequent sections).

### Barriers to facility-based HIV services – getting to the clinic

Consistently across all three countries, one of the most significant barriers to accessing facility-based HIV services was related to physical accessibility to and of HIV services facilities. Across all impairment types, many participants mentioned the lack of accessible physical infrastructure (poor roads, lack of sidewalks and ramps, inability to use public transportation) as well as the social and emotional trauma of being taunted by other riders or the driver, or having to pay extra for their crutch or wheelchair on a bus. Particularly in Uganda, many participants frequently spoke about how taxi drivers did not pick them up or they were turned away because of their disability. In all three countries, some spoke of travelling with an assistant to help them but admitted that this brought additional complications due to difficulty of finding someone prepared to give up their time and be publicly seen with a person with HIV, and the additional transport costs required.

We, the blind, we have challenge – most of our guides do not want to guide us to the areas where the services are offered simply because they fear the community associating them with the HIV/AIDS. (Male, blind, 58, Uganda)

Another difficulty is that as a result of the long queues at [Clinic X], we as people who are blind are being denied to be escorted by friends and family. They refuse saying when we go, we'll spend the whole day at the clinic just for nothing. (Male, blind, 40, Zambia)

For many, regardless of type of impairment, attending clinic visits with an assistant also presented a challenge to maintaining confidentiality about their HIV status, particularly when picking up medications or attending consultations.

Sometimes we get to be escorted by family members or friends due to the fact that we can't manage moving alone. So you'll find that the one who escorted you gets to know all your HIV status details and yet information is supposed to be confidential. (Male, physically disabled, 40, Zambia)

Despite the many challenges, a number of participants in Zambia and Uganda reported positive experiences with non-governmental organizations (NGOs) that provided home-based care and outreach services. In particular, participants reported outreach by non-clinical support workers was essential in helping them receive medications, counselling and health education without having to visit a facility.

We the disabled get services through caregivers like [NGO1], and some come through to our communities and give us information concerning our health. … And if found positive, they tell us how to live positively. (Female, physically disabled, 25, Zambia)

We get these services from [NGO2], and if we are unable to get there, we have peer counselors who carry drugs and come down to the grassroots where we are and provide the services to us. (Female, physically disabled, 50, Uganda)

### Facility-level barriers – within the clinic

Once within the health facility, participants reported varying experiences with regard to how services accommodated their impairment-specific needs. In all three countries, many participants indicated that although they had not been directly refused services because of their disability, the challenges they had encountered at the health facilities (most often at government facilities) were so numerous and discouraging that they often ended up forgoing HIV treatment or seeking services elsewhere (e.g. at private facilities or from traditional healers). Disability-specific inaccessibility at health facilities that were mentioned often in all countries included lack of sign language interpreters and Braille or large-print materials, inaccessible toilets and lack of ramps and wide doors for wheelchairs.

In Uganda, a number of participants reported several ways in which healthcare facilities and providers had recognized and addressed their needs; there were no such experiences reported in Ghana and Zambia. Participants indicated that some health facilities were beginning to respond to their needs by improving infrastructure and making accessible information available. Many participants mentioned improved accessibility to some government facilities including construction of ramps and availability of printed HIV-related information resources in large font and pictures.

After a series of advocacy for provision of HIV/AIDS care and treatment to the blind and physically disabled, the government responded partially to reduce the gap which was affecting the blind and physically disabled. … some of these included building of ramps in hospitals for the physically disabled to easily get the service they need. (Male, blind, 49, Uganda)

The lack of skills and sensitivity among healthcare providers emerged strongly from participants in all countries regardless of impairment type. Many participants felt that they were missing out on critical information about how to take care of themselves as a person living with HIV, including taking medications correctly. Deaf participants felt that it was difficult to receive counselling and instructions for taking and adhering to medications. Facilities lacked informational materials in accessible format, and several participants spoke about their desire for more information on living positively.

As positive deaf, services are problematic because there are no interpreters, so it makes us miss important information instructions on how to take medication which is a health risk. (Male, deaf, 48, Zambia)

While some deaf persons were able to receive healthcare information through written resources (e.g. leaflets, posters), they acknowledged that this was not possible for illiterate persons, which is common among persons with disabilities due to barriers accessing education.

Participants commonly reported that healthcare providers, anticipating communication challenges, frequently gave priority to people without disabilities, leading to extended wait times and consequently medication stock-outs by the end of the day.

For us the blind people when we go to those hospitals, they make us sit down and wait and at the end day they don't provide you with any services, eventually they tell you there is no medicine. (Female, blind, 50, Uganda)

When doctors see a deaf person approaching, there is communication breakdown and therefore do not attend to us. Instead they call hearing people and attend to them. … so we are turned down. We feel depressed and demoralized so we just go home and sleep. (Male, deaf, 48, Zambia)

### Economic barriers

Many participants pointed out that they face excessive economic challenges due to costs associated with travel to clinics, clinical services and food to support the increased nutritional needs of people on ART.

We have only one challenge of being poor. … the medicine requires us to eat something, so you see that many will become reluctant and not take the medicine simply because they do not have the money to buy the food to accompany the medicine. So they end up not taking the medicine at a regular basis as prescribed by the doctors. (Male, physically disabled, 38, Uganda)

Challenges associated with limited financial resources is especially hard for persons with disabilities; many participants talked about how persons with disabilities are more often unemployed, less educated and live in poverty compared with those without a disability. Many participants, particularly from Ghana, frequently discussed the interconnected linkages between disability and lack of education and illiteracy.

Lacking education, they are at an extreme disadvantage in comprehending existing HIV prevention messages. I think the main issue is most of us are not mainly educated, so these words [about HIV] when they are mentioned, if you don't get someone to explain it to you, then you are lost. (Female, 30, deaf, HIV positive, Accra, Ghana)

However, there were instances mentioned of economic support, all of which were from Uganda and Zambia. Some participants in Uganda, through local NGOs and community-based organizations (CBOs), reported receiving additional supportive services that help them improve their own livelihoods such as the formation of income-generating activities.

We as HIV positive and physically disabled people often get groups through which we can access services such as counseling, medicines, and knowledge. For example, here in Jinja, we have [CBO]; … we may engage in poultry farming starting from 2 chickens to find means of how to help ourselves. That is why we are thankful to [CBO], and other NGOs which have given us pigs, seedlings for agricultural farming which we rear and gain money and also get food for our personal nutrition in the long run. Hence these groups help us collectively advocate for the services we need. (Male, physically disabled, 58, Uganda)

### Stigma related to HIV and disabilities

In all three countries, participants reported experiencing multiple dimensions of stigma from multiple sources, compounding each other to result in social isolation and being cut off from sources of critical information and services. The findings from this study related to the multiple sources of stigma among persons with disabilities, including those living with HIV, have been reported elsewhere [26]. Briefly, the dual stigma of HIV and disability as well as the internalized stigma (i.e. feeling ashamed because of their disability and HIV status) discouraged people from HIV testing due to fear of judgment from others and concern about who will take care of them. This was pervasive in all three countries. These overlapping stigmas are the paramount underlying reason for late HIV diagnosis, sub-optimal attendance at health clinics for ART services and lack of family and community support. Stigmatizing attitudes were rampant in the community as well as at health facilities. Many participants reported that they experienced stigmatizing attitudes from other patients and even healthcare providers when accessing HIV-related services.

[We] are neglected and segregated by the medical people. Some say we smell. You try very much to seek for his or her attention; the medical person just passes by you so when you go back, you fail to the guts or energy to go back to the hospital because of the way you were treated the day before. … [we] lose the morale of getting treatment from the facilities. (Male, physically disabled, 38, Uganda)

To alleviate some of the issues around stigma, some participants in Uganda talked about how instrumental social support from other persons with disabilities living with HIV was in allowing them to deal more effectively with stressors related to living with HIV because there was a sense that others face similar challenges or will be there to help them if necessary.

We form our groups as we [people with disabilities] who are HIV positive such that other persons like us cannot think they are alone and this helps to build their spirits and motivate them to living healthy lives. (Female, physically disabled, 50, Uganda)

### Access to services by sex

Most female participants felt they had more challenges accessing services compared with males because of their gender roles. Across all three countries, female participants mentioned household and childcare responsibilities and having less money than men as the challenges in seeking healthcare.

For a man it is easier because we women have a lot to take care of at the home and would not have enough time to go get services. (Female, physically disabled, 25, Zambia)

While there was evidence of differential access to healthcare, most participants felt that men and women with disabilities were not treated differently by providers based on their sex: “We are treated equally. They don't say you are a man or a woman” (Female, deaf, 30, Ghana). However, a few female participants in Zambia mentioned longer wait times for women: “Men are treated first. Women wait in a queue until they are done with them [men], then they start calling names of women” (Female, deaf, 54, Zambia).

## Discussion

Through the voices of persons with disabilities living with HIV, this study highlighted specific challenges and facilitators for persons with disabilities living with HIV in accessing HIV services. They encounter many challenges in accessing HIV testing and continued care and treatment services. These barriers exist at many levels: individual (e.g. lack of accessible HIV information), psychosocial (e.g. stigma), economic (e.g. poverty) and health systems (e.g. provider attitudes and skills, inaccessible physical infrastructure). While some of the barriers are similar to those experienced by HIV-positive persons without disabilities (e.g. HIV-related stigma, long queues at health facilities), these barriers are amplified for persons with disabilities.

The barriers discussed in this paper mirror findings from other studies on persons with disabilities in sub-Saharan Africa [2,4,9,11,13–18]. However, this study emphasizes the struggles faced specifically by persons with disabilities living with HIV in accessing HIV testing and obtaining HIV care and treatment services, which may ultimately have a negative impact on HIV treatment outcomes. We found that the “double burden” of being HIV positive and having a disability and the associated stigma lead to delays in accessing essential services such as HIV testing due to fear of results and potential consequences of a positive result. In addition, many delayed HIV diagnosis until they felt sick and upon diagnosis, they did not want to seek care and treatment due to the many challenges they faced getting to the facilities as well as within the facilities. Challenges at point of care highlighted in this study include lack of sensitization and skills among healthcare workers to work with this population and lack of accessible infrastructure which significantly inhibited persons with disabilities living with HIV from obtaining the services they need including information on correct medication usage, adherence and how to live positively. This has major implication for HIV treatment outcomes for persons with disabilities living with HIV as late HIV diagnosis and late initiation (or lack of use) of ART is associated with greater morbidity and mortality.

There is a need to make services accessible to the disabled and sensitize health workers to provide services to persons with disabilities. Further, programmes need to reach out to persons with disabilities for testing and treatment initiation. For example, testing as well as ART can be provided through DPOs or at home through home-based services as shown by some programmes in Zambia and Uganda. Such interventions are part of a compendium of best practices in HIV programming for persons with disabilities [27]. In addition, interventions should not only be targeted at improving services and infrastructure such as provision of sign interpreters and accessible materials or provision of outreach services, programmes must also address stigma reduction and gender equity within the larger community to reduce the stigma associated with HIV and disability and the harmful gender norms that impede the access of women with disabilities to access health services.

Despite the evidence of many challenges in accessing HIV services, this study also found favourable experiences emerging from Uganda and Zambia, resulting from actions initiated by NGOs and DPOs and supportive national policies. Although progress may be relatively slow, Zambia and in particular Uganda serve as examples in supporting and implementing policies and programmes to provide persons with disabilities living with HIV with tailored HIV services. Reports of positive experiences from persons with disabilities living with HIV in Uganda, even within government facilities, are not surprising given that Uganda has one of the most progressive National Strategic Plan for HIV/AIDS with regard to persons with disabilities with specific guidelines for operationalization [19,21]. Supportive policies at the national level as in the case of Uganda and Zambia where there has been systematic inclusion of persons with disabilities in the national HIV planning efforts are likely the reason for the evidence of favourable programming for persons with disabilities in these countries. Such policies pave the way for inclusive services within mainstream health facilities and other efforts by DPOs and NGOs (e.g. home-based care, income generation activities and support groups).

## Limitations

Although selection of participants according to different impairments enabled us to capture a range of experiences, the study sample was small and may not be representative of persons with disabilities living with HIV in sub-Saharan Africa. However, the remarkable similarities in the barriers across the three countries despite the different stages of HIV response to persons with disabilities suggest that there are some common challenges across sub-Saharan African settings. Further, the sample comprised persons with disabilities who were linked in some way to DPOs and many had basic schooling. Thus, the sample may be more resourced and connected than others with disabilities. This may potentially have biased the results to more favourable reports as those not linked to services may likely experience and report negative experience to a greater degree than what is reported here. However, the participants in this study spoke not only about their own experiences as a person with disabilities living with HIV but also about others in similar circumstances. In addition, the use of FGDs, as opposed to in-depth interviews, may have biased the findings as the sample consisted of those who were comfortable with openly discussing their experiences as an HIV-positive person. Those not comfortable being part of a group discussion may represent a subset with greater challenges to accessing services given their discomfort with disclosure. Finally, this study did not include persons with intellectual or developmental disabilities due to ethical concerns. However, this does not indicate that they are free from HIV risk. There is evidence that it is a population at risk for HIV [12,28].

## Conclusions

The barriers reported in this study have major implications for the HIV treatment outcomes of persons with disabilities living with HIV and for reaching the UNAIDS 90-90-90 HIV treatment targets [29]. Changes are needed at every level to ensure persons with disabilities have access to HIV services including provision of accessible services, infrastructure and information; formation of support groups for persons with disabilities; changing harmful attitudes around disabilities; HIV and gender norms within the community and in health facilities; and outreach and home-based interventions to mitigate accessibility barriers.
